# Clinical and Molecular Epidemiology of Human Parainfluenza Viruses 1–4 in Children from Viet Nam

**DOI:** 10.1038/s41598-018-24767-4

**Published:** 2018-05-01

**Authors:** Martin Linster, Lien Anh Ha Do, Ngo Ngoc Quang Minh, Yihui Chen, Zhu Zhe, Tran Anh Tuan, Ha Manh Tuan, Yvonne C. F. Su, H. Rogier van Doorn, Mahesh Moorthy, Gavin J. D. Smith

**Affiliations:** 1 Programme in Emerging Infectious Diseases, Duke-NUS Medical School, Singapore, Singapore; 2 Oxford University Clinical Research Unit-Viet Nam, Ho Chi Minh City, Vietnam; 3 Murdoch’s Children Research Institute, Melbourne, Australia; 4 Children’s Hospital 1, Ho Chi Minh City, Vietnam; 5 Children’s Hospital 2, Ho Chi Minh City, Vietnam; 6 Centre for Tropical Medicine and Global Health, Nuffield Department of Clinical Medicine, University of Oxford, Oxford, UK; 7 Department of Clinical Virology, Christian Medical College, Vellore, India; 8 Duke Global Health Institute, Duke University, Durham, North Carolina USA

## Abstract

HPIVs are serologically and genetically grouped into four species that account for up to 10% of all hospitalizations due to acute respiratory infection in children under the age of five. Genetic and epidemiological data for the four HPIVs derived from two pediatric cohorts in Viet Nam are presented. Respiratory samples were screened for HPIV1–4 by real-time PCR. Demographic and clinical data of patients infected with different HPIV were compared. We used a hemi-nested PCR approach to generate viral genome sequences from HPIV-positive samples and conducted a comprehensive phylogenetic analysis. In total, 170 samples tested positive for HPIV. HPIV3 was most commonly detected in our cohort and 80 co-detections of HPIV with other respiratory viruses were found. Phylogenetic analyses suggest local endemic circulation as well as punctuated introductions of new HPIV lineages. Viral gene flow analysis revealed that Viet Nam is a net importer of viral genetic diversity. Epidemiological analyses imply similar disease severity for all HPIV species. HPIV sequences from Viet Nam formed local clusters and were interspersed with sequences from diverse geographic regions. Combined, this new knowledge will help to investigate global HPIV circulation patterns in more detail and ultimately define more suitable vaccine strains.

## Introduction

Human parainfluenza viruses (HPIVs) are members of the family *Paramyxoviridae* and circulate globally as four species. They are classified into two genera based on their genome, HPIV1 and HPIV3 as genus *Respirovirus*, and HPIV2 and HPIV4 as genus *Rubulavirus*^1^.

HPIV infection can manifest as both acute upper respiratory infections (AURI) and acute lower respiratory infections (ALRI)^2^. Although HPIV infections are generally self-limiting, they occasionally require hospitalization and may lead to mortality. Infections with HPIV are second only to respiratory syncytial virus (RSV) as a cause of ALRI-associated hospitalization in children under the age of five years^3^.

Specific clinical diagnoses have been attributed to individual HPIV species, e.g. HPIV1 and HPIV2 infection with laryngotracheobronchitis (croup) and HPIV3 with bronchiolitis. These clinical associations, however, are neither predictive of a certain HPIV species nor mutually exclusive. Low- and middle-income countries have a high burden of disease due to severe ALRI with the highest number of HPIV infections among young children. High-risk populations include individuals with chronic respiratory conditions, prematurely-born infants, and the immunosuppressed. HPIV3, in particular, infects younger children after waning of maternal antibodies^4^. Nonetheless, country-specific descriptions of clinical profiles for individual HPIV species are largely lacking^5^. Clinical severity has been attributed to the ability of the virus to efficiently replicate in the upper respiratory tract prior to spread to the lower respiratory tract^2,6^. Whereas the majority of HPIV infections occurs in children, these viruses can infect individuals from all age groups. Consequently, they likely form one gene pool with endemic circulation across all ages.

Factors contributing to successful transmission include shedding of the virus at sufficiently high titers, from the upper respiratory tract, environmental stability of the virion and ability to initiate infection upon contact with a new host. HPIVs replicate abundantly in the tracheal epithelium facilitating transmission from the upper respiratory tract and are likely highly infectious, as was demonstrated for a closely related murine virus^7,8^. Transmission of HPIVs is by close contact and short-range transmission by respiratory droplets with minimal aerosol transmission^9^. HPIV also retains its infectivity on inanimate objects for up to 10 hours^10^ and its incubation period as found in volunteer studies was estimated at 3–6 days^11^. No specific antivirals or vaccines to treat and prevent HPIV infection are currently available.

Periodicity and magnitude of HPIV epidemics is likely determined by susceptibility to infection, partial cross-protective immunity and differences in virus transmissibility^12–15^. Disease presentation and severity varies between HPIV species, geographic locations and over time^16^. However, our understanding of the molecular epidemiology and global transmission of HPIV is limited since sequence information to date is mainly restricted to the hemagglutinin-neuraminidase (HN) gene, while full genome data is sparse and biased towards the Americas.

The present study describes species-specific clinical presentation, the genetic variability and HPIV circulation in Viet Nam. The outcome of RSV infection in hospitalized children under 2 years of age presenting with acute lower respiratory infection (ALRI) in Ho Chi Minh City was described previously^17^. A second study was conducted enrolling children under 16 years of age presenting with acute respiratory infection (ARI) to outpatient departments. Respiratory samples from both cohorts that were positive for HPIV were sourced into the current study for sequencing purposes. Using Bayesian phylogenetic methods, we also infer the importation of HPIV into Viet Nam from viral sequence data and report high viral diversity within the country.

## Methods

### Patients and Samples

Samples were sourced from two previous acute respiratory infection (ARI) cohorts among in- and outpatients, that were conducted at Children’s Hospital 1 and 2 in Ho Chi Minh City, Viet Nam during years 2009 and 2010.

Detailed information on patient enrolment, sample collection and clinical parameters for the inpatient study are provided in Do *et al*.^17^. Briefly, children under 2 years of age were eligible if admitted for a lower respiratory tract infection with an onset of symptoms less than 5 days prior to recruitment. Patients with uncomplicated non-respiratory or non-infectious respiratory causes for hospitalisation were excluded. In the outpatient study children under 16 years of age, living in Ho Chi Minh City, and with a clinical diagnosis of acute respiratory infection (defined as presenting with cough, sore throat, runny nose or nasal congestion for less than 5 days as the chief complaint) with no underlying illness except asthma were enrolled at the outpatient clinic of Children’s Hospital 1.

Three types of respiratory specimens (nasal swabs, throat swabs, and nasopharyngeal aspirates) were collected on admission by trained personnel using standard operating procedures, and were placed in viral transport medium^17^. Specimens were kept at 4 °C for a maximum of 24 h and then aliquoted and stored at −80 °C until further processing. RNA was extracted and viral nucleic acid was detected as described previously^17–19^. The studies were approved by the Scientific and Ethical Committee of the Hospital for Tropical Diseases, the Institutional Review Boards of Children’s Hospital 1 and 2 and the Oxford University Tropical Research Ethical Committee under the approval numbers 44.08 for the inpatient and 31.08 for the outpatient study. Written informed consent was obtained from parents or legal guardians of all children enrolled in the study.

### PCR Amplification and Sequencing

Complementary DNA (cDNA) was prepared from HPIV-positive samples as previously described^20^ and shipped to Duke-NUS for PCR amplification and Sanger sequencing. For sequencing, HPIV-positive samples were further amplified in a hemi-nested PCR approach with newly designed species-specific primers targeting overlapping regions of the viral genome (Table S2). Briefly, the first-round PCR was conducted in 5 or 6 separate reactions and amplified an ‘outer’ PCR product of about ~3 kb collectively covering the entire genome of HPIV (~15 kb for HPIV1–3, ~17 kb for HPIV4). These initial PCR products were further amplified in a second-round PCR yielding 2 separate overlapping ‘inner’ products of 1.5–1.8 kb. Amplicons were gel-purified and sequenced with PCR primers. If required, PCRs and sequencing reactions using additional primers were performed to achieve full genome coverage. Low quality reads were manually curated, primer sequences were removed and contigs were assembled in Geneious version 7.0^21^. Nucleotide alignments of the HN gene of all HPIV species and whole genome sequences of HPIV3 were created using MAFFT^22^.

### Phylogenetic Analysis

All available HN gene sequences of the 4 HPIV species were downloaded from the NIAID Virus Pathogen Database and Analysis Resource (ViPR)^23^ and annotated with sample collection date and country. Temporal phylogenies of individual HPIV species were inferred in BEAST v1.8.3^24^ using a strict clock, the HKY85 substitution model, and a constant coalescent tree prior. MCMC chains were run for 100 million steps with sampling every 10,000 generations. Run convergence was confirmed in Tracer v1.6 after 10% burn-in removal^25^. Maximum clade credibility (MCC) trees were generated using Tree Annotator v1.7^26^. The phylogeny of HPIV3 was further inferred from available whole-genome sequences. All phylogenetic trees were visualized using FigTree v1.4.2 and annotated with the following geographic locations if present in the dataset: Africa (including the Middle East), East Asia (excluding Viet Nam), Europe, North America, Oceania, South America, South Asia and Viet Nam.

For HPIV3, we quantified migration in and out of Viet Nam using HN gene flow for the years 2009 and 2010. Available sequence data between years 2009 and 2010 was grouped into Viet Nam and Others, and the number of discrete state transitions or Markov jumps to or from Viet Nam were estimated in BEAST v1.8.3. Mean rates of migration in and out of Viet Nam were inferred using an asymmetric substitution model with the Bayesian Stochastic Search Variable Selection (BSSVS) option. Two independent runs of 100 million generations were combined using LogCombiner v1.8.3 after removal of appropriate burn-in and the mean migration rates were extracted from the combined log file.

### Epidemiological Analysis

We analysed the clinical characteristics of HPIV species from the two studies from which our samples were sourced. Demographics (age, sex) and clinical metadata (duration of illness at presentation, clinical symptoms pulse, respiratory rate, total episode duration, ICU admission, clinical diagnosis and disease outcome) recorded during the original studies were used. For each of the studies, we assessed the variability of parameters (age, duration of illness, pulse, and respiratory rate) across HPIV species with a Kruskal-Wallis test with a Dunn post-test. Proportions of demographic and clinical variables were compared between HPIV species with a *Chi*-squared test with Yates’ correction. The clinical diagnosis at presentation and follow-up were classified as upper respiratory infection (rhinitis, rhinopharyngitis or laryngitis) or bronchiolitis/pneumonia or any lower respiratory infection (either bronchitis, croup, bronchiolitis or pneumonia). A *p*-value of <0.05 was considered significant. All statistical analyses were performed on GraphPad Prism 5.0d for Mac software (GraphPad Software, La Jolla, California USA).

### Data availability

All data generated or analysed during this study are included in this published article (and its Supplementary Information files). Sequence data generated on this study has been deposited in the NCBI repository (https://www.ncbi.nlm.nih.gov/nuccore). Any additional information is available from the authors on request.

## Results

A total of 170 cDNA samples tested positive for HPIV (N = 19, 24, 85 and 42 for HPIV1–4, respectively). Of these, 90 patient samples tested positive for only a single HPIV species while 11 were co-infections of two HPIV species; HPIV2 and HPIV3 (N = 1), HPIV2 and HPIV4 (N = 1), HPIV3 and HPIV4 (N = 9) (Table 1). The remaining 69 (41%) HPIV-positive samples also tested positive for other respiratory viruses. In general, HPIV3 and HPIV4 were more commonly associated with viral co-infections, with Rhinovirus/Enterovirus most frequently co-detected, being present in 47 of 170 (28%) of HPIV infections. Notably, bocavirus was found as a co-infection with each HPIV species, and was found in 15 of 170 (9%) HPIV-positive samples.

**Table 1:** Detection of HPIV and other viral pathogens among the outpatient and inpatient cohorts.

	HPIV1	HPIV2	HPIV3	HPIV4
Mono-infection	10/4/14	11/4/15	26/16/42	10/9/19
Co-infection	2/3/5	3/6/9	30/13/43	18/5/23
HPIV2	0		—	—
HPIV3	0	0/1/1		—
HPIV4	0	0/1/1	9/0/9	
HAdV	1/0/1	0	5/2/7	1/0/1
HRSV	0	1/0/1	0/1/1	0
HMPV	1/0/1	0	4/1/5	2/1/3
RV/EV	0/1/1	2/3/5	18/7/25	13/3/16
HCoV	0	1/0/1	4/2/6	3/0/3
HPeV	0	0/2/2	1/0/1	0
HBoV	1/2/3	1/1/2	4/4/8	1/1/2
**Total**	12/7/19	14/10/24	56/29/85	28/14/42

Number of positives for outpatients/inpatients/total. No HPIV-positive samples tested positive for influenza A or B virus. HPIV, human parainfluenza virus; HAdV, human adenovirus; HRSV, human respiratory syncytial virus; HMPV, human metapneumovirus; RV, rhinovirus; EV, enterovirus; HCoV, human coronavirus; HPeV, human parechovirus; HBoV, human bocavirus.

We first examined the clinical presentation of HPIV in the outpatient study that recruited children under 16 years with ARI. In this study, the median age and gender distribution of infected children and frequency of clinical presentation (fever, cough, sore throat, runny nose and nasal congestion) as well as vital signs (pulse and respiratory rate) and duration of illness at presentation were similar between HPIV species (*p* > 0.05). For all the HPIV species, presentation as an illness with wheeze was more common than with crepitations. Next, we compared the clinical diagnosis at presentation and follow-up. The proportion of pneumonia or bronchiolitis at presentation was 4/12 (33%), 1/14 (7%), 21/56 (38%), 6/28 (21%) for HPIV1–4, respectively. Further, the proportion presenting as lower respiratory infection was high both at presentation [10/12 (83%), 8/14 (57%), 39/56 (70%) and 18/28 (64%)] and follow-up [10/12 (83%), 9/12 (75%), 36/52 (69%), 16/28 (57%)] for HPIV 1–4, respectively, indicating a high morbidity and poor resolution of symptoms.

The inpatient study consisted of children under 2 years of age with a diagnosis of ALRI^17^. The age, gender distribution and clinical presentation were similar between viral species. For all 4 HPIV species, at least one clinical sign of ALRI (rapid respiratory rate or intercostal in-drawing) was present in the majority of children [HPIV1 6/7 (86%), HPIV2 10/10 (100%), HPIV3 25/29 (86%), HPIV4 14/14 (100%)]. Bronchiolitis was more commonly seen with HPIV1, 2 and 4 (5/7, 71%; 5/8, 63%; 12/14, 86%; respectively) compared to HPIV3 (15/28, 54%). Complete clinical recovery at 7 days post-admission was infrequent for HPIV1 and HPIV4 (1/7, 2/14, 14% each, respectively) compared to HPIV2 and HPIV3 (4/8, 50% and 13/28, 46%, respectively) (p > 0.05). The duration of the ALRI episode as inferred by a telephonic interview at 4–6 months, was similar for all HPIV species (p > 0.05). Oxygen therapy at admission was only required for a subset of patients infected with HPIV3 (2/29, 7%) and HPIV4 (1/14, 7%), while only the two HPIV3 infected patients were admitted to intensive care.

We were able to generate sequences from 71 (42%) of the available 170 cDNA samples from both studies. In total, we obtained 293 viral gene sequences, wherein 201 were above 90% total gene coverage. For co-infections, sequences were obtained from the HPIV species with the lower C_q_ value. The individual virus names, sampling dates, sequenced viral genes and corresponding accession numbers are listed in Supplementary Table 1.

**Figure 1:**
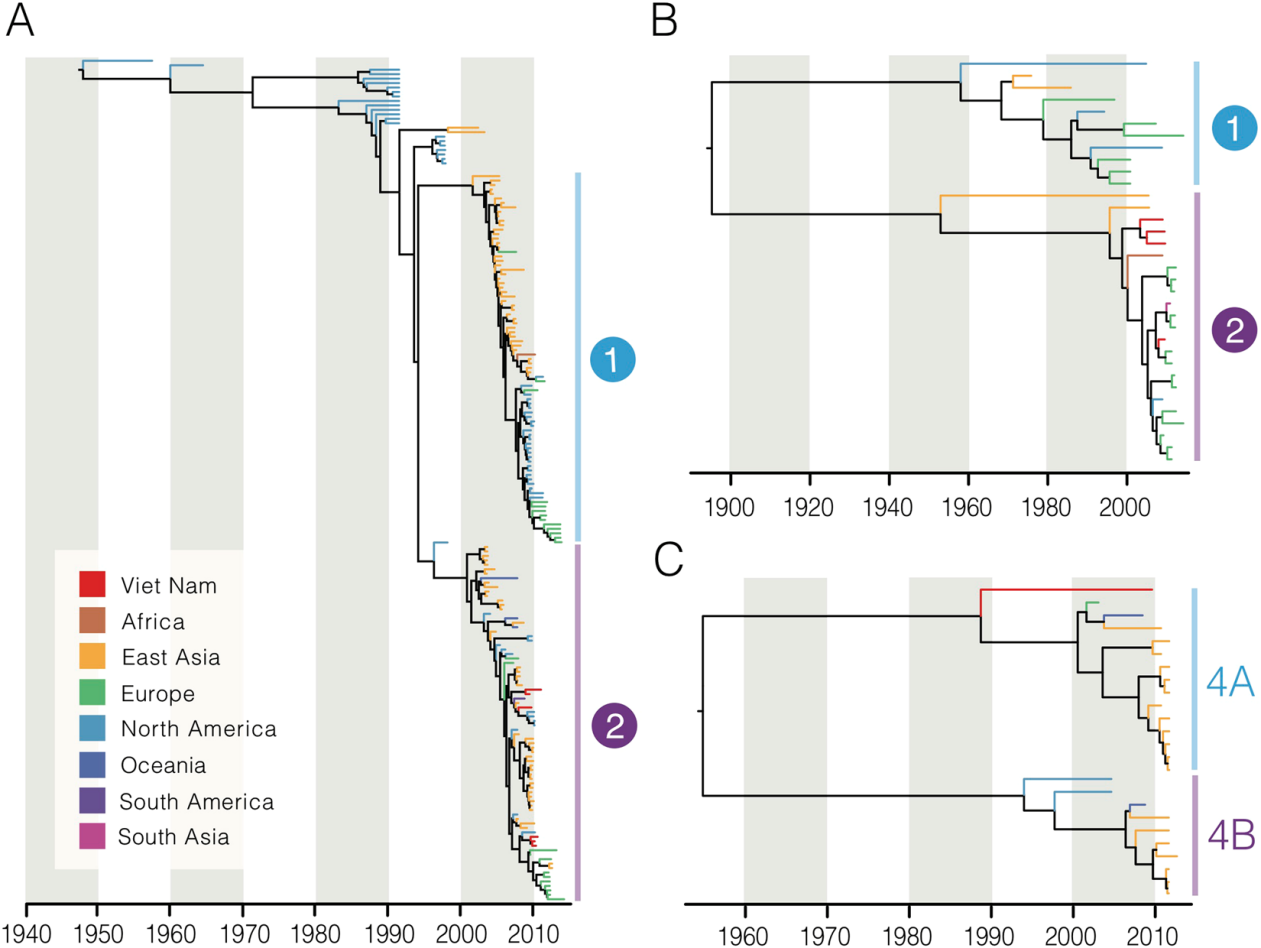
Dated trees of the HN gene of HPIV1 (**A**), HPIV2 (**B**), and HPIV4 (**C**); terminal branches are colored by geographical region of virus isolation. (**A**) HPIV1 global phylogeny. Clades 1 and 2 correspond to clusters identified previously in the literature. (**B**) HPIV2 phylogeny. Representative clades are labelled for discussion purposes. (**C**) The HPIV4 phylogeny comprises two clades corresponding to previously described subspecies 4A and 4B.

We reconstructed the temporal phylogeny using the newly generated full-length HN gene of HPIV1–4 (N = 6, 4, 30 and 1, respectively) and publicly available data (N = 182, 30, 489 and 24, respectively). Results indicated that HPIV1 has been circulating in humans since the 1940’s, with time to the most recent common ancestor (TMRCA) estimated as 1948 (95% highest posterior density (HPD): 1944–1952) (Table 2). Since the mid-1990s, HPIV1 has diverged into two distinct monophyletic lineages (clades 1 and 2) that co-circulate worldwide. All Vietnamese viruses sequenced within this study clustered in clade 2 along with viruses from Argentina, Japan and USA (Fig. 1A). Limited genetic data is available for HPIV2 and HPIV4 and this may influence the estimation of dates and these results must therefore be interpreted with caution. The mean TMRCA for HPIV2 was estimated as 1896 (95% HPD: 1850–1925). HPIV2 also diverged into two clades with clade 1 viruses predominant in the 1980s until they were superseded by clade 2 viruses during the early 2000s (Fig. 1B). All Vietnamese HPIV2 viruses sequenced from this study, as well as all sequences obtained after the year 2000 from Asia, belong to clade 2. The mean TMRCA of the HPIV4 viruses was estimated at 1955 (95% HPD: 1938–1968), while the TMRCAs for HPIV4A and HPIV4B were estimated as 1989 (95% HPD: 1981–1994) and 1994 (1989–1998), respectively. The single Vietnamese HPIV4 sequence grouped with HPIV4A subspecies viruses but was relatively distant and in a basal position to other known HPIV4A virus sequences.

**Table 2:** TMRCAs and nucleotide substitution rates with 95% highest posterior density (HPD) intervals reported for each dataset.

Dataset	TMRCA (mean)	Lower 95% HPD	Upper 95% HPD	Substitution rate (×10^−3^ subs/site/yr)	Lower 95% HPD	Upper 95% HPD
HPIV1 HN	1947.98	1943.83	1951.60	1.197	1.052	1.344
HPIV2 HN	1895.89	1849.76	1924.83	0.320	0.219	0.417
HPIV3 HN	1943.37	1938.30	1947.90	0.839	0.772	0.911
HPIV3 Viet Nam clade*	2005.38	2004.20	2006.47	—	—	—
HPIV4 HN	1954.85	1937.54	1968.47	1.683	1.226	2.126

**Figure 2:**
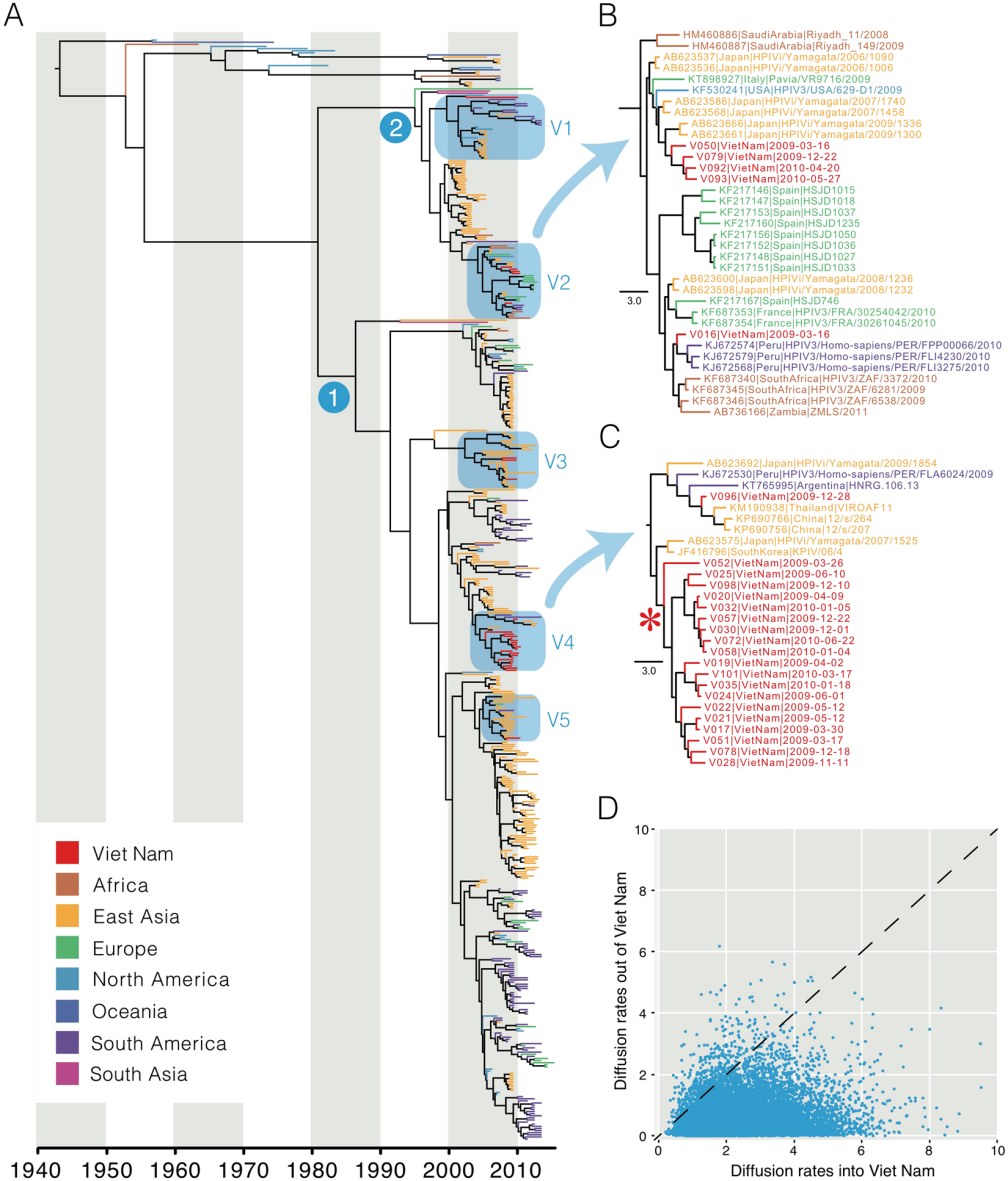
HPIV3 HN global phylogeny with terminal branches colored by geographical region of virus isolation (**A**). Nodes 1 and 2 indicate the common ancestors of lineages previously described in the literature. Clades containing the Vietnamese sequences generated in this study are labelled V1–V5, with clade V2 (**B**) and clade V4 (**C**) shown in detail. Within V4, a large monophyletic clade comprising sequences from Viet Nam is indicated with an asterisk. (**D**) Scatter plot of mean migration rates of HPIV3 HN in and out of Viet Nam in 2009–2010. The diagonal line indicates identical mean rates of migration in and out of Viet Nam.

An asterisk denotes the monophyletic clade of HPIV3 viruses from Viet Nam indicated in Fig. 2C.

The mean TMRCA of HPIV3 was estimated at 1943 (95% HPD: 1938–1948), which is similar to the age of HPIV1 (Table 2). HPIV3 has also diverged into two major monophyletic clades (clades 1 and 2 in Fig. 2). In marked contrast to other HPIVs, the HPIV3 phylogeny exhibits more extensive lineage diversification in the 2000s, with some indication of continuous genetic drift, indicated by a ladder-like phylogeny^27^, that is more evident within the clade 1 than clade 2 (Fig. 2A). Viral lineage turnover is also present, whereby older virus strains become extinct and are replaced by newer strains. Clade 1 viruses also demonstrated greater diversification of multiple independent lineages and inter-continental mixing of the viruses than clade 2 viruses. Our results also show that the HPIV3 infections in Viet Nam during 2009–2010 resulted from at least five independent virus introductions that are highlighted as clades V1–V5 in Fig. 2A. The viruses detected in Viet Nam were generally closely related to viruses from East Asia, although clades comprised viruses from diverse geographic areas (for examples see Fig. 2B and C). In clades V2 and V4 there is one cluster each of HPIV3 viruses from Viet Nam that were detected over a period of 14–15 months, suggesting sustained endemic circulation. The mean TMRCA of clade V4 was estimated as 2005 (95% HPD: 2004–2006) suggesting that these viruses may have been circulating in Viet Nam for at least 5 years. However, we cannot exclude the possibility that this may be due to inadequate global sampling and sequencing of HPIV3 viruses. We also reconstructed the HPIV3 phylogeny using 24 newly generated whole genomes and 138 publicly available genomes. The whole-genome HPIV3 phylogeny was largely congruent with the HN phylogeny, showing multiple clades of viruses that circulate globally (Supplementary Fig. 1).

We further performed phylogeographic analysis to investigate the extent of HPIV3 migration into and out of Viet Nam during 2009–2010. Our results show that the total diffusion rates of HPIV3 into Viet Nam from all other areas are significantly greater than from Viet Nam into all other regions . This indicates that Viet Nam was not acting as a source population for epidemics elsewhere but was a net importer of HPIV3 diversity in 2009–2010.

## Discussion

This study reaffirms the described clinical presentation of HPIV species^2^. In both cohorts, HPIV-infected patients presented within the clinical spectrum of both AURI and ALRI. Characteristic clinical features could not be attributed to a specific HPIV species. Among outpatients, all four HPIV species predominately manifested with ALRI (bronchitis, bronchiolitis, pneumonia). This is remarkable in the case of HPIV4, which was previously known to cause mild clinical illness^28^. The observation of lower respiratory tract involvement resulting from infection with all HPIV species at initial consultation, with only incomplete resolution of symptoms at follow-up, is an indication of high disease severity in this cohort. In the inpatient cohort, which in contrast to the outpatient cohort used clinically manifesting ALRI as an inclusion criterion, two cases of HPIV3 infection resulted in admission to the intensive care unit, possibly indicating enhanced pathogenicity of HPIV3 over the other species as suggested previously^1,4,29^, although the episode duration was similar for all HPIV species.

All HPIV species have been detected in diverse geographical regions and several circulation patterns have been suggested^30,31^. In our study, viral co-detections were common among samples positive for all HPIV species. Our rate of viral co-infections of 41% is within previous reports and highlights the importance for screening for several viral pathogens simultaneously^32^. Rhino-/Enteroviruses were most commonly found in combination with HPIV, possibly due to longer duration of shedding with the former and/or frequent orofecal transmission in paediatric patients^33^. Notably, co-detection of bocavirus was found among all HPIV species and in both cohorts. Furthermore, the complete absence of influenza virus co-infection in both cohorts is remarkable. Human adenovirus, human respiratory syncytial virus, human metapneumovirus, seasonal coronaviruses, and human parechovirus were only incidentally detected in combination with HPIV. The concurrent detection of HPIV3 and HPIV4 occurred in nine instances in the outpatient cohort, suggesting this combination to be rather common. HPIVs are usually detected throughout the year, but enhanced epidemic transmission probably occurs during the colder months of the year. Fry *et al*. described an interaction of HPIV1 and HPIV3 detections in the US^30^. In our study, we found co-detection of HPIVs year-round, however, no distinct peak was observed. This is likely due to the study design as the original studies among in- and out-patients were conducted over a limited time span.

We here report a high number of co-detections, which is in line with previous acute respiratory infection studies^32,34^. However, we were unable to identify specific clinical symptoms associated with individual HPIV species, and future studies employing an inclusive case definition are needed to identify risk factors for severe disease in cases of HPIV infection and co-infection.

Several factors might have contributed to the fact that not all detected HPIVs could be amplified and sequenced. First, suboptimal primer design is likely due to the limited number of available sequences per HPIV species available in public repositories and unknown sequence diversity; second, we did not retest positive qPCR results allowing for false-positive test results; and third, the RNA might have degraded after their initial collection and during long-term storage and shipping.

Phylogenetic analysis for HPIV2 and HPIV4 was restricted due to low sequence numbers from Viet Nam and globally. Two groups of HPIV1 co-circulate globally but only restricted diversity was detected in this study. Viet Nam viruses belong to clade 2 and are most closely related to virus sequences from Argentina, Japan and USA. In contrast, HPIV3 sequences from Viet Nam belonged to multiple clades and grouped with viruses from diverse geographical regions. Multiple introductions of HPIV3 into Viet Nam were observed in 2009 and 2010, and in two cases these may have resulted in sustained endemic transmission, while clades comprised of viruses from different geographical areas suggests frequent transmission between those areas. In addition, phylogeographic analysis indicated that Viet Nam was a net importer of HPIV3 diversity. While other viruses such as influenza A/H3N2 viruses and RSV circulate globally^35–38^, this has not been demonstrated for HPIV, but is a probable scenario. However, the current lack of sequence data, particularly from large population centres, renders it difficult to draw firm conclusions on the transmission of these viruses at any temporal or spatial scale without additional sampling from diverse geographical regions.

## Electronic supplementary material

Supplementary Materials
