# Supplementary material for: Clinical and Molecular Epidemiology of Human Parainfluenza Viruses 1–4 in Children from Viet Nam
Source: Sci Rep. 2018 May 1;8:6833. doi: 10.1038/s41598-018-24767-4 (PMC5931535; doi:10.1038/s41598-018-24767-4)
Supplement: Supplementary file 1 — Supplementary Materials [file 41598_2018_24767_MOESM1_ESM.pdf]

Supplementary Table 1. Name of virus, sampling date, HPIV genes sequenced per sample and accession numbers respectively. Parentheses indicate partial genome sequences.

Virus Name	Sampling Date	Genes	Accession Number
HPIV1/VietNam/002/2009	2009-02-10	(HN)	MH006614
HPIV1/VietNam/004/2009	2009-03-09	(P/C), (M), (F), (HN), (L)	MH006615
HPIV1/VietNam/005/2009	2009-04-02	(HN)	MH006616
HPIV1/VietNam/006/2009	2009-11-23	(N), (P/C), M, F, HN, L	MH006617
HPIV1/VietNam/061/2009	2009-06-10	(N), (P/C), M, (F), HN, (L)	MH006618
HPIV1/VietNam/062/2010	2010-01-27	(F), HN, (L)	MH006619
HPIV1/VietNam/063/2010	2010-04-20	(N), (P/C), M, F, HN, (L)	MH006620
HPIV1/VietNam/064/2010	2010-10-04	(F), (HN), (L)	MH006621
HPIV1/VietNam/067/2010	2010-06-02	(HN)	MH006622
HPIV2/VietNam/008/2009	2009-07-30	N, V/P, M, F, HN, L	MH006623
HPIV2/VietNam/014/2010	2010-01-27	HN, (L)	MH006624
HPIV2/VietNam/015/2010	2010-02-04	(F), HN, (L)	MH006625
HPIV2/VietNam/068/2010	2010-03-03	(N), (M), (F), (L)	MH006626
HPIV2/VietNam/069/2010	2010-03-18	N, (V), (M), (F), HN, L	MH006627
HPIV2/VietNam/073/2010	2010-03-16	(HN), (L)	MH006628
HPIV3/VietNam/001/2010	2010-01-14	N, P/C/D, M, F	MH006629
HPIV3/VietNam/016/2009	2009-03-16	N, (P/C/D), M, F, HN, (L)	MH006630
HPIV3/VietNam/017/2009	2009-03-30	N, P/C/D, M, F, HN, L	MH006631
HPIV3/VietNam/018/2009	2009-03-31	N, M, F, (HN), (L)	MH006632
HPIV3/VietNam/019/2009	2009-04-02	N, P/C/D, M, F, HN, L	MH006633
HPIV3/VietNam/020/2009	2009-04-09	N, P/C/D, M, F, HN, L	MH006634
HPIV3/VietNam/021/2009	2009-05-12	N, P/C/D, M, F, HN, L	MH006635
HPIV3/VietNam/022/2009	2009-05-12	N, (P/C/D), M, F, HN, L	MH006636
HPIV3/VietNam/023/2009	2009-06-01	N, P/C/D, (L)	MH006637
HPIV3/VietNam/024/2009	2009-06-01	N, P/C/D, M, F, HN, L	MH006638
HPIV3/VietNam/025/2009	2009-06-10	N, P/C/D, M, F, HN, L	MH006639
HPIV3/VietNam/028/2009	2009-11-11	N, (P/C/D), F, HN, L	MH006640
HPIV3/VietNam/029/2009	2009-11-24	N, (P/C/D), M, F, HN, L	MH006641
HPIV3/VietNam/030/2009	2009-12-01	N, (P/C/D), M, F, HN, L	MH006642
HPIV3/VietNam/032/2010	2010-01-05	N, P/C/D, M, F, HN, (L)	MH006643
HPIV3/VietNam/035/2010	2010-01-18	HN, (L)	MH006644
HPIV3/VietNam/050/2009	2009-03-16	HN, (L)	MH006645
HPIV3/VietNam/051/2009	2009-03-17	N, P/C/D, M, F, HN, L	MH006646
HPIV3/VietNam/052/2009	2009-03-26	N, P/C/D, M, F, HN, (L)	MH006647
HPIV3/VietNam/054/2009	2009-11-10	HN, (L)	MH006648
HPIV3/VietNam/057/2009	2009-12-22	(HN), (L)	MH006649
HPIV3/VietNam/058/2010	2010-01-04	HN, (L)	MH006650
HPIV3/VietNam/059/2010	2010-01-06	(F), (L)	MH006651
HPIV3/VietNam/060/2010	2010-01-11	(L)	MH006652
HPIV3/VietNam/072/2010	2010-06-22	HN, (L)	MH006653
HPIV3/VietNam/074/2009	2009-11-05	N, P/C/D, M, F, HN, L	MH006654

HPIV3/VietNam/075/2009	2009-11-27	N, P/C/D, M, F, (L)	MH006655
HPIV3/VietNam/076/2009	2009-12-09	N, P/C/D, (L)	MH006656
HPIV3/VietNam/077/2009	2009-12-11	(HN), (L)	MH006657
HPIV3/VietNam/078/2009	2009-12-18	N, P/C/D, M, F, HN, L	MH006658
HPIV3/VietNam/079/2009	2009-12-22	N, P/C/D, M, F, HN, (L)	MH006659
HPIV3/VietNam/081/2009	2009-12-22	N, P/C/D, M, F, (HN), (L)	MH006660
HPIV3/VietNam/082/2009	2009-12-28	N, P/C/D, (F), (L)	MH006661
HPIV3/VietNam/083/2010	2010-01-11	N, P/C/D, M, (L)	MH006662
HPIV3/VietNam/084/2010	2010-01-12	P/C/D, M, F	MH006663
HPIV3/VietNam/085/2010	2010-01-20	N	MH006664
HPIV3/VietNam/086/2010	2010-01-28	N, P/C/D, M, F, HN, (L)	MH006665
HPIV3/VietNam/087/2010	2010-03-19	N, (P/C/D), M, (L)	MH006666
HPIV3/VietNam/088/2010	2010-03-24	N, P/C/D	MH006667
HPIV3/VietNam/089/2010	2010-03-28	N, P/C/D, F, (HN), (L)	MH006668
HPIV3/VietNam/090/2010	2010-04-06	P/C/D, (L)	MH006669
HPIV3/VietNam/092/2010	2010-04-20	(N), P/C/D, M, F, HN, (L)	MH006670
HPIV3/VietNam/093/2010	2010-05-27	N, P/C/D, M, F, HN, (L)	MH006671
HPIV3/VietNam/094/2010	2010-05-28	N, P/C/D, M, F, HN, L	MH006672
HPIV3/VietNam/095/2010	2010-12-01	N, (P/C/D), M, F, (HN), (L)	MH006673
HPIV3/VietNam/096/2009	2009-12-28	N, P/C/D, M, F, HN, L	MH006674
HPIV3/VietNam/097/2010	2010-03-19	P/C/D, M, F, (HN)	MH006675
HPIV3/VietNam/098/2009	2009-12-10	N, P/C/D, M, F, HN, L	MH006676
HPIV3/VietNam/099/2010	2010-01-05	(HN), (L)	MH006677
HPIV3/VietNam/100/2010	2010-01-22	(HN), (L)	MH006678
HPIV3/VietNam/101/2010	2010-03-17	N, P/C/D, M, F, HN, (L)	MH006679
HPIV3/VietNam/102/2010	2010-12-28	N, M, F, (L)	MH006680
HPIV4/VietNam/044/2009	2009-05-26	(HN), (L)	MH006681
HPIV4/VietNam/045/2009	2009-07-22	(V/P), (HN), (L)	MH006682
HPIV4/VietNam/046/2009	2009-12-08	(HN)	MH006683
HPIV4/VietNam/112/2009	2009-05-28	(N), (V/P), HN, (L)	MH006684

Supplementary Table 2. Primer sequences used to amplify and sequence HPIV genomes.

Fragment	PIV type and primer name	Primer sequence	Amplicon size
F1.1	HPIV1_F1_77F<br>HPIV1_F1.1_1575R	ARGGACAAGTCACAGACATTTGA<br>CCTTCGTGCAATCTTGTTTTCTA	1498
F1.2	HPIV1_F1.2_1364F<br>HPIV1_F1_2971R	CYATAGAAGTGGCAATTGATCATACA<br>TCTTCTACTTTATTCTCAAGTGATTTTTCA	1607
F2.1	HPIV1_F2_2892F<br>HPIV1_F2.1_4598R	CAAGTCATGTATTTGCTAAGCGCA<br>CCAGATGACTAGATTCATATGTGGA	1706
F2.2	HPIV1_F2.2_4350F<br>HPIV1_F2_5976R	AAGCTCAACTTCATGGTACACCTA<br>GCCTGGTATWTCTGATAAAATTGGTATT	1626
F3.1	HPIV1_F3_5750F<br>HPIV1_F3.1_7292R	GCTCCAATCTTGGGACTATAGGAG<br>GTTGCATGACTTCTCTATTAATTGTGT	1542
F3.2	HPIV1_F3.2_7155F<br>HPIV1_F3_9059R	GAGACAATCACAGAATTAATCAGACA<br>AYCGGATAAGGTTCAAAAGTGTAAC	1904
F4.1	HPIV1_F4_8752F<br>HPIV1_F4.1_10370R	TTAGGGTTAATGCCTGCYTATTTG<br>RTCATTTAACCATTCTCCTGATTCTACA	1618
F4.2	HPIV1_F4.2_10199F<br>HPIV1_F4_11652R	TCTATCACCTAGGAAAGCAGCAT<br>TGCTGGATCACCTATATTTCTGACA	1453
F5.1	HPIV1_F5_11471F<br>HPIV1_F5.1_13099R	GCCAACAGGTATGTATATCATTAGGAAT<br>GCTGTGCATATACTAGTTGCTCT	1628
F5.2	HPIV1_F5.2_12965F<br>HPIV1_F5_14558R	GTTAATCTATGATCCTGATCCTCTCA<br>GGTATGCAATCCTGATCACACTATAAT	1593
F6.1	HPIV1_F6_14274F<br>HPIV1_F6.1_15061R	CTTGTGATCTCAACGGACAAAGA<br>AGCTTCTTATCTGCATCTTTTATCCTA	787
F6.2	HPIV1_F6.2_14809F<br>HPIV1_F6_15610R	TAGTTGAGAAAGCCAAAGTTCATGAT<br>CCAGACAAGAGTTTAAGAAATATCGATATA	801
F1.1	HPIV2_F1_8F<br>HPIV2_F1.1_1536R	GGAGAATAGATGGCATCGTTATATGA<br>TTGATCGTTGTCAATTCTGTCCT	1528
F1.2	HPIV2_F1.2_1332F<br>HPIV2_F1_3666R	AGACCTCACTGCAACCATAT<br>CAAATCCATACGTATTGATGAAAGTT	2334
F2.1	HPIV2_F2_3508F<br>HPIV2_F2.1_5243R	ATCCAACTTCACCCAGTCAATC<br>TGGATTGAATTGAAGATGCAAGATT	1735
F2.2	HPIV2_F2.2_4896F<br>HPIV2_F2_6885R	TTCAATCAAAGATAAGATCACTCATGT<br>CGGAAAATGATTCTACATGTCCTT	1989
F3.1	HPIV2_F3_6655F<br>HPIV2_F3.1_8154R	TTAAGGTGTCGTAACGTCTC<br>ACTTGATAGGACGGTACCCATT	1499
F3.2	HPIV2_F3.2_7985F<br>HPIV2_F3_9714R	CAAGTCATGATGGGTGCAGAA<br>TTAAGTTGTAGACACCCATATACTAAAG	1729
F4.1	HPIV2_F4_9594F<br>HPIV2_F4.1_11135R	TAGGGCCAAGGATAGATAGATTGTT<br>TATCTCCTTGAACCATGCTCATT	1541
F4.2	HPIV2_F4.2_10921F<br>HPIV2_F4_12609R	ATCTACATTATATGTTGGTGATCCATTC<br>TCAAGTGCATCATTCCAGTTCA	1688
F5.1	HPIV2_F5_12441F<br>HPIV2_F5.1_14058R	ATCCACCCATCAGAGTTCCATA<br>TCGAGAGATTCTGGATGTGGAA	1617
F5.2	HPIV2_F5.2_13880F<br>HPIV2_F5_15601R	CAAATTAGAGAATCAGACGAAGGA<br>TCATTGTTGTCATAATTCTTCGC	1730
F1.1	HPIV3_F1_54F<br>HPIV3_F1.1_1715R	TTAGGATTAAAGACATTGASTRGAAGG<br>CTTTAATCCTAAGTTTTTCTTATTTMTTAT	1661
F1.2	HPIV3_F1.2_1600F	CAAACAGAACRAAYCAGGAYGAAATAG	2131

	HPIV3_F1_3731R	CTTTAATCCTAAGTTTTTCTTATTTGTATATTG	
F2.1	HPIV3_F2_3330F	TATATCGACATGCAGGARAYACAYTRGA	1566
	HPIV3_F2.1_4895R	TTGACYTCTTTTRTCCTAAGTTTTTGAT	
F2.2	HPIV3_F2.2_4714F	CAAGAGTRGATGCAATTTTCCAACCYTC	2032
	HPIV3_F2_6746R	TAACTTTACTCCTAAGTTTTTTRTAATTTTAAT	
F3.1	HPIV3_F3_6362F	AAAGAAGGAACYCTTRCATTCTAYACA	1486
	HPIV3_F3.1_7847R	CATTYTCATTTATTGGRTGTTCAAGACCT	
F3.2	HPIV3_F3.2_7622F	AAGTYGATGAAAGATCAGAYTATGCATCAT	1803
	HPIV3_F3_9425R	TTGTAATTTBGGATCTAAYTTRGCRCATG	
F4.1	HPIV3_F4_9280F	TGATACATCCAGAATTGGTTTTGATATT	1903
	HPIV3_F4.1_11182R	GCTTGAGGAAGAATTCTCCCATCA	
F4.2	HPIV3_F4.2_11011F	ACAGAGTTAAGAARGAGATAGTTTATAA	1861
	HPIV3_F4_12871R	ACATCTTTGAGYGGGTCTTTATCAT	
F5.1	HPIV3_F5_12746F	AGAAGATGAGTGTTGTATTAAAGAAAG	1884
	HPIV3_F5.1_14629R	TCACTAGGTTCCATTATAGTACAATA	
F5.2	HPIV3_F5.2_14427F	ACRTAYTTGATTGGRGATGATGATGT	966
	HPIV3_F5_15392R	GGCTYAARGATAAAGGTTAVGATATA	
F1.1	HPIV4A_F1_122F	MRGACCTTAAATAAAGTAGCTCAT	1387
	HPIV4A_F1.1_1590R	TTGTGATCTGAGTTGGTTGAATCAAT	
F1.2	HPIV4A_F1.2_1305F	AACTTGGAATAACTGATGAAGACAGAGT	1770
	HPIV4AB_F1_3075R	CAAGTGTAATTGTATTGTCTTGATCAAC	
F2.1	HPIV4AB_F2_2949F	GAYCCTGGAGTCCCATCAAAAGTA	1619
	HPIV4A_F2.1_4568R	ATGATCTCACAACCTATGGACCAAA	
F2.2	HPIV4A_F2.2_4160F	UACCUAGACCUGUACUAAACUU	1881
	HPIV4AB_F2_5987R	CATYACTGAAGCTGCCGAGA	
F3.1	HPIV4A_F3_5683F	AGGCAGTTCGAGATCTTGCAAA	1055
	HPIV4A_F3.1_6738R	CUGUCAAUAUUAAUAAGAUGCAGAGU	
F3.2	HPIV4A_F3.2_7313F	AACACACTTCTCAGCCCTGA	1401
	HPIV4AB_F3_8714R	AAATGCACTCTGTATAAGTCTC	
F4.1	HPIV4A_F4_8485F	ATATCGCACCAGGGGTAGG	1731
	HPIV4A_F4.1_10216R	TTCTTACCTGGCTCCAATTGATTG	
F4.2	HPIV4A_F4.2_9969F	AAGCAATGAGGAACAAAAAAGATAGA	1463
	HPIV4A_F4_11432R	CAAWYAGTGATATCCATTTCCAGTA	
F5.1	HPIV4A_F5_11049F	AUGAUGUUUUCUCAGUUGAAGAGAGU	2017
	HPIV4A_F5.1_13066R	AGGACGTCTCATGATATTTGTTAAG	
F5.2	HPIV4A_F5.2_12768F	AUGUGGCAAUAAGACUAAAUAUAUUCAUGG	1801
	HPIV4A_F5_14569R	TGATCCAAGTAAGTATTTAAGAATTCACT	
F6.1	HPIV4A_F6_14159F	ACAUGGAAUGCAUUGCCUAUUAA	1592
	HPIV4A_F6.1_15751R	AGUGACACCAUUCCAUAAACACUU	
F6.2	HPIV4A_F6.2_15536F	AGTGGTGCTTCTATGACATTGATA	1569
	HPIV4A_F6_17105R	TAAGAGAGATCGATACCGAACGATA	

Supplementary Figure 1. Dated trees of HPIV sequences with full isolate names indicated per type, HPIV1 (A), HPIV2 (B), HPIV3 (C), HPIV4 (D), corresponding to Figures 1A, 1B, 2A and 1C respectively in the main text.

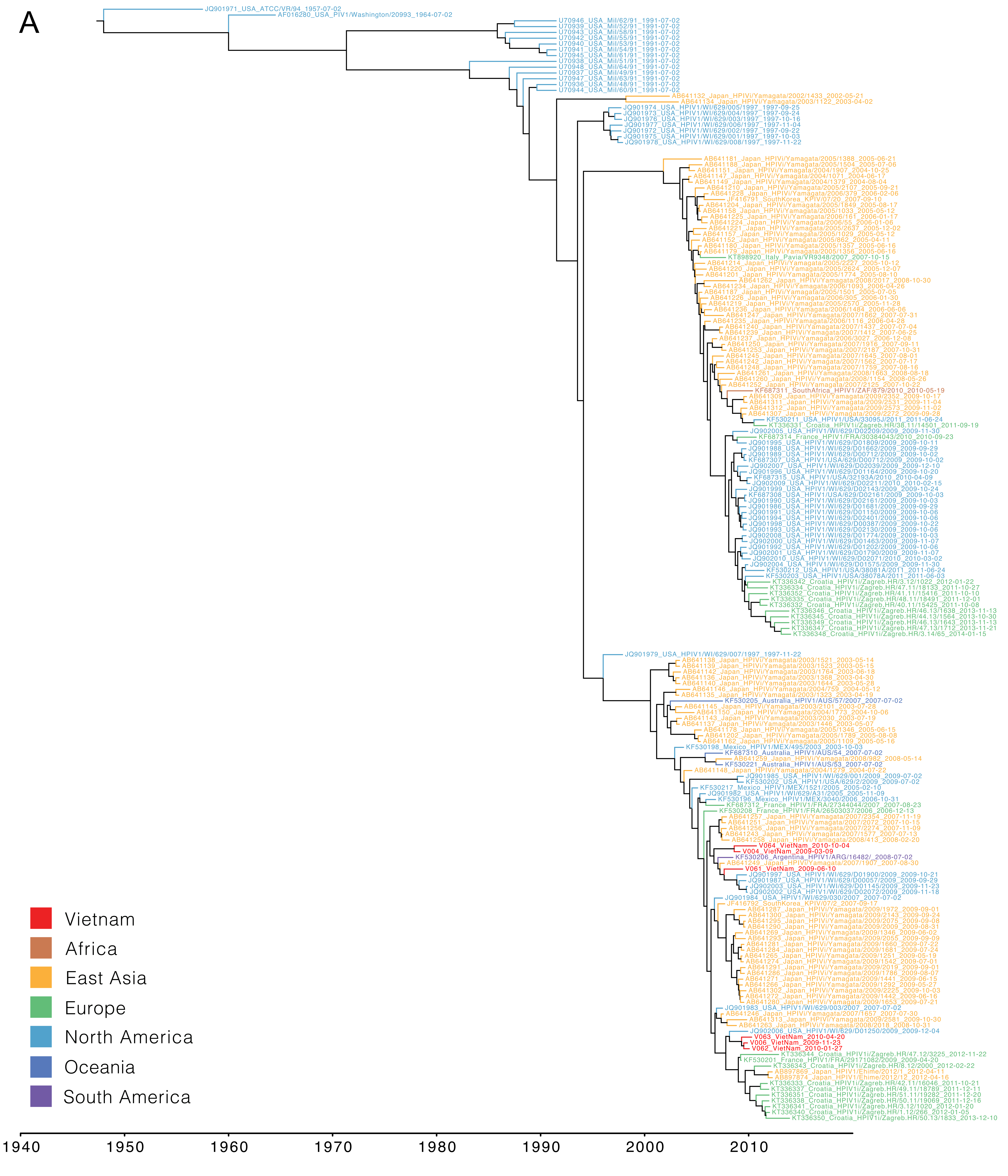
A
Vietnam
Africa
East Asia
Europe
North America
Oceania
South America
1940
1950
1960
1970
1980
1990
2000
2010

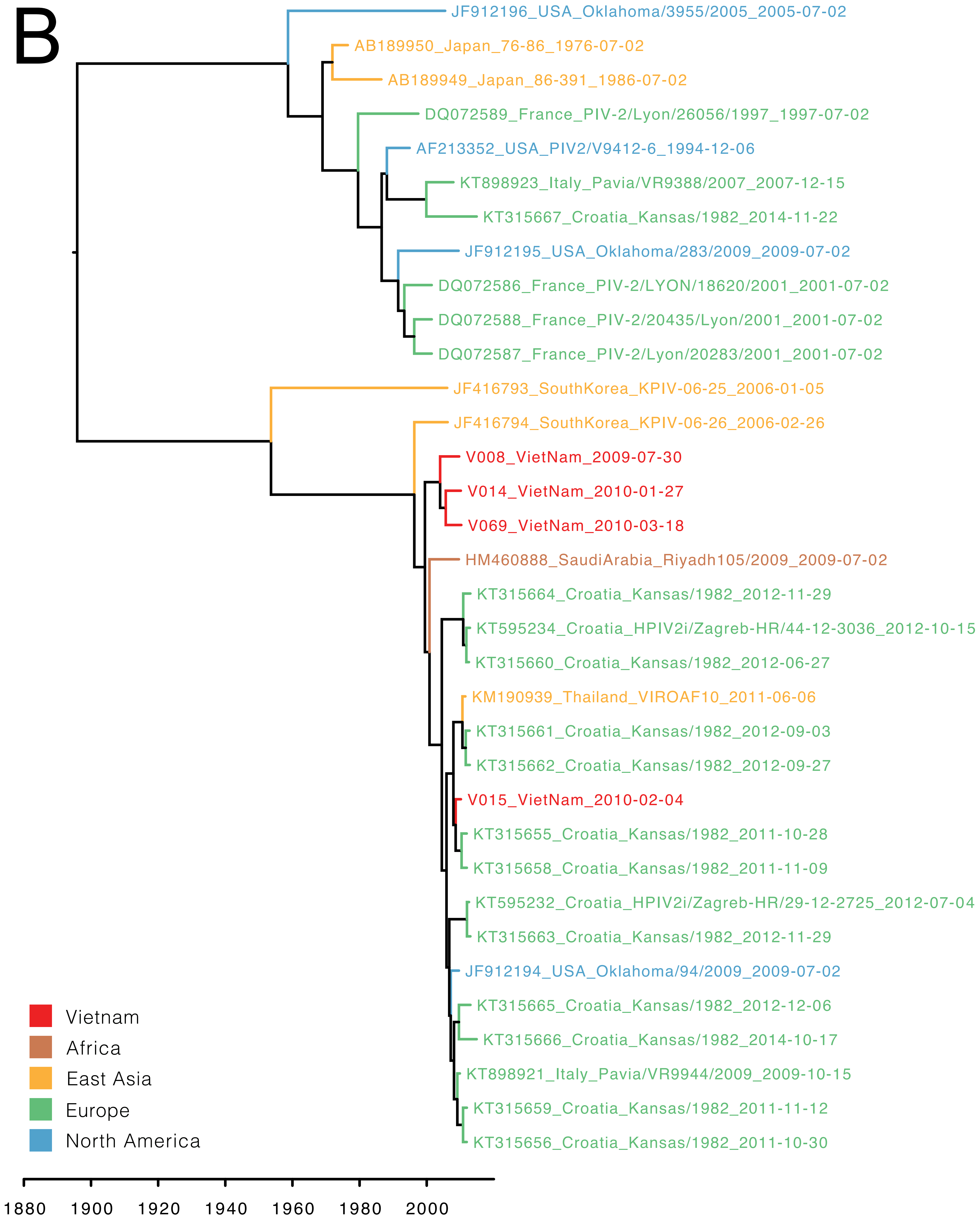
B
JF912196_USA_Oklahoma/3955/2005_2005-07-02
AB189950_Japan_76-86_1976-07-02
AB189949_Japan_86-391_1986-07-02
DQ072589_France_PIV-2/Lyon/26056/1997_1997-07-02
AF213352_USA_PIV2/V9412-6_1994-12-06
KT898923_Italy_Pavia/VR9388/2007_2007-12-15
KT315667_Croatia_Kansas/1982_2014-11-22
JF912195_USA_Oklahoma/283/2009_2009-07-02
DQ072586_France_PIV-2/LYON/18620/2001_2001-07-02
DQ072588_France_PIV-2/20435/Lyon/2001_2001-07-02
DQ072587_France_PIV-2/Lyon/20283/2001_2001-07-02
JF416793_SouthKorea_KPIV-06-25_2006-01-05
JF416794_SouthKorea_KPIV-06-26_2006-02-26
V008_VietNam_2009-07-30
V014_VietNam_2010-01-27
V069_VietNam_2010-03-18
HM460888_SaudiArabia_Riyadh105/2009_2009-07-02
KT315664_Croatia_Kansas/1982_2012-11-29
KT595234_Croatia_HPIV2i/Zagreb-HR/44-12-3036_2012-10-15
KT315660_Croatia_Kansas/1982_2012-06-27
KM190939_Thailand_VIROAF10_2011-06-06
KT315661_Croatia_Kansas/1982_2012-09-03
KT315662_Croatia_Kansas/1982_2012-09-27
V015_VietNam_2010-02-04
KT315655_Croatia_Kansas/1982_2011-10-28
KT315658_Croatia_Kansas/1982_2011-11-09
KT595232_Croatia_HPIV2i/Zagreb-HR/29-12-2725_2012-07-04
KT315663_Croatia_Kansas/1982_2012-11-29
JF912194_USA_Oklahoma/94/2009_2009-07-02
KT315665_Croatia_Kansas/1982_2012-12-06
KT315666_Croatia_Kansas/1982_2014-10-17
KT898921_Italy_Pavia/VR9944/2009_2009-10-15
KT315659_Croatia_Kansas/1982_2011-11-12
KT315656_Croatia_Kansas/1982_2011-10-30
Vietnam
Africa
East Asia
Europe
North America
1880
1900
1920
1940
1960
1980
2000

C

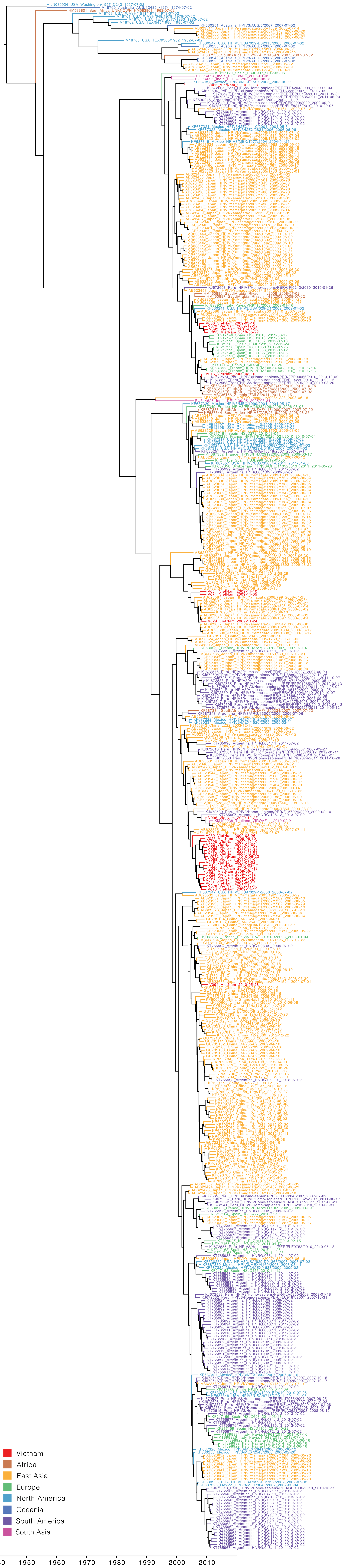

Vietnam
Africa
East Asia
Europe
North America
Oceania
South America
South Asia
1940
1950
1960
1970
1980
1990
2000
2010

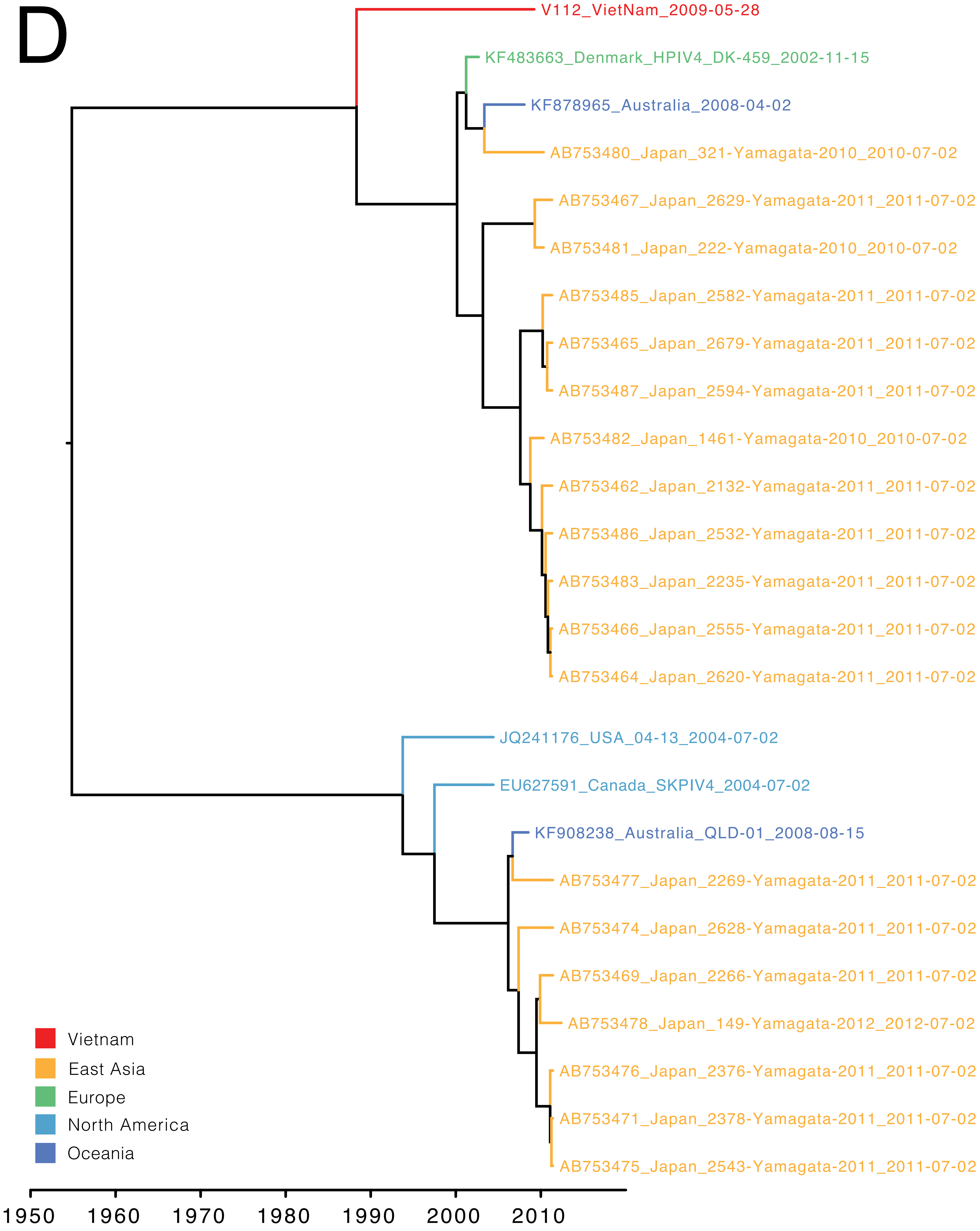
D
V112_VietNam_2009-05-28
KF483663_Denmark_HPIV4_DK-459_2002-11-15
KF878965_Australia_2008-04-02
AB753480_Japan_321-Yamagata-2010_2010-07-02
AB753467_Japan_2629-Yamagata-2011_2011-07-02
AB753481_Japan_222-Yamagata-2010_2010-07-02
AB753485_Japan_2582-Yamagata-2011_2011-07-02
AB753465_Japan_2679-Yamagata-2011_2011-07-02
AB753487_Japan_2594-Yamagata-2011_2011-07-02
AB753482_Japan_1461-Yamagata-2010_2010-07-02
AB753462_Japan_2132-Yamagata-2011_2011-07-02
AB753486_Japan_2532-Yamagata-2011_2011-07-02
AB753483_Japan_2235-Yamagata-2011_2011-07-02
AB753466_Japan_2555-Yamagata-2011_2011-07-02
AB753464_Japan_2620-Yamagata-2011_2011-07-02
JQ241176_USA_04-13_2004-07-02
EU627591_Canada_SKPIV4_2004-07-02
KF908238_Australia_QLD-01_2008-08-15
AB753477_Japan_2269-Yamagata-2011_2011-07-02
AB753474_Japan_2628-Yamagata-2011_2011-07-02
AB753469_Japan_2266-Yamagata-2011_2011-07-02
AB753478_Japan_149-Yamagata-2012_2012-07-02
AB753476_Japan_2376-Yamagata-2011_2011-07-02
AB753471_Japan_2378-Yamagata-2011_2011-07-02
AB753475_Japan_2543-Yamagata-2011_2011-07-02
Vietnam
East Asia
Europe
North America
Oceania
1950
1960
1970
1980
1990
2000
2010
